# Dementia awareness and risk perception in middle-aged and older individuals: baseline results of the MijnBreincoach survey on the association between lifestyle and brain health

**DOI:** 10.1186/s12889-019-7010-z

**Published:** 2019-06-03

**Authors:** Irene Heger, Kay Deckers, Martin van Boxtel, Marjolein de Vugt, KlaasJan Hajema, Frans Verhey, Sebastian Köhler

**Affiliations:** 1 Alzheimer Centrum Limburg, School for Mental Health and Neuroscience, Department of Psychiatry and Neuropsychology, Maastricht University, Dr. Tanslaan 12, 6229 ET, Maastricht, The Netherlands; 2 GGD Zuid-Limburg, Postbus 33, 6400 AA Heerlen, The Netherlands

**Keywords:** Dementia literacy, Awareness, Lifestyle, Risk factors, Prevention, Brain health, Health promotion

## Abstract

**Background:**

The total number of people with dementia is increasing worldwide, due to our aging society. Without a disease-modifying drug available, risk reduction strategies are to date the only promising way to reduce dementia incidence in the future. Substantial evidence exists that lifestyle factors contribute to the risk of dementia, such as physical exercise, mental activity and (non-)smoking. Still, most people seem unaware of a relationship between lifestyle and brain health. This paper investigates dementia literacy and knowledge of modifiable risk and protective factors of dementia in a Dutch population-based sample.

**Methods:**

An online-survey was carried out among 590 community-dwelling people between 40 and 75 years old in the Province of Limburg, the Netherlands. The total group comprises both of a provincial sample (*n* = 381) and a sample of three specific districts within the province (*n* = 209). Dementia awareness and knowledge about 12 risk and protective factors was assessed with items derived from the British Social Attitudes (BSA) survey, supplemented with custom items developed by the research team.

**Results:**

The majority of participants (56%) were unaware of a relationship between lifestyle and dementia risk. Most individuals identified low cognitive activity, physical inactivity and unhealthy diet as dementia risk factors. Particular gaps in knowledge existed with regard to major cardiovascular risk factors such as hypertension, hypercholesterolemia and coronary heart disease. Although the level of awareness varied by age and level of education, most people (70%) were eager to learn more about the topic of brain health, and indicated to be interested in using eHealth (54%) to measure or improve brain health.

**Conclusions:**

Most people still are unaware of the relation between lifestyle and brain health, indicating the need for public health campaigns. Increasing awareness in the general population about the presence of modifiable dementia risk and protective factors is a crucial first step prior to implementation of preventative measures. Targeting specific subgroups, such as individuals with low socioeconomic status and low health literacy, is essential for the reach and effect of a prevention campaign. Outcome of this study was the rationale for an awareness campaign in The Netherlands, called “*MijnBreincoach*” (“MyBraincoach”).

**Electronic supplementary material:**

The online version of this article (10.1186/s12889-019-7010-z) contains supplementary material, which is available to authorized users.

## Background

Dementia is a syndrome characterized by cognitive dysfunction leading to interference with daily life activities. Alzheimer’s disease and cerebrovascular damage are the most common underlying causes of dementia, with many patients showing evidence for both [1, 2]. Dementia is one of the most common causes of disability and mortality among older individuals and has considerable psychosocial effects for both the person with the diagnosis and for relatives and informal caregivers [3–5]. According to the current estimates, 47 million people are living with dementia worldwide. It is expected that this number will triple to 131 million by 2050, with the largest relative increase in low-andmiddle-income countries [6]. In the Netherlands, 270,000 people had one form of dementia in 2017, and this figure is expected to more than double by 2055 [7]. The associated global societal economic costs are predicted to rise steadily, making dementia a trillion-dollar disease in 2018 [6, 8].

Despite extensive global research, there is to date no curative treatment for the common forms of dementia [9], but several risk factors have been identified. Besides non-modifiable risk factors, such as age, sex and genetics, there is good support for modifiable risk factors as contributors to the risk of developing dementia in later life [10]. Recent estimations suggest that one in three dementia cases may be attributable to common modifiable risk factors [11]. Several healthy living behaviours have been identified, e.g. regular physical exercise, high mental activity and adequate blood pressure control [12]. Development of effective risk reduction strategies to prevent dementia, or delay its onset, is recently receiving increasing attention in research and policy [4, 13–17]. With regard to the question when to start targeting these risk and protective factors, it seems that the earlier in life, the better. Research shows that the predictive value of these factors for cognitive impairment and dementia in the very old (85+) is poor [18]. Interventions aimed at promoting healthy lifestyle might therefore be most effective in younger stages of life, such as midlife [18–21].

Yet, there seems to be a relative lack of dementia risk awareness in the general public, resulting in major gaps of knowledge on dementia in general, and on the relation between lifestyle and brain health in particular. A recent systematic review showed that almost 50% of all respondents perceive dementia as an inevitable and non-preventable part of living [22]. An Australian survey from 2009 showed that about one-third of all respondents believed that nothing can be done about the risk of dementia, and respondents most often could not identify common risk factors [23]. The recent British Social Attitudes (BSA) survey showed that dementia is a major public health concern for most people, but their knowledge of dementia risk factors was poor. Only 1% of the respondents identified the seven risk and protective factors mentioned in the survey correctly and 22% could not identify any of the factors. In addition, more than half of participants agreed with the statement “there is nothing one can do to lower one’s dementia risk” or said they do not know [2].

In order to identify specific target groups and address their needs and wishes in future strategies for dementia prevention, the aim of this study was to evaluate dementia literacy and knowledge of dementia risk and protective factors in a well-defined geographical region: the province of Limburg in the South of the Netherlands. We report on the findings of two different samples, as well as differences between certain subgroups (e.g. gender, age, level of education) with regard to dementia risk awareness. Findings were the rationale of an awareness campaign about the relationship between lifestyle and brain health in the province of Limburg, called *“MijnBreincoach”* (“MyBraincoach”).

## Methods

### Study design and recruitment

This cross-sectional study is part of *MijnBreincoach,* a public health campaign of the Alzheimer Centrum Limburg, which is part of the Maastricht University Medical Centre (MUMC+) in the Netherlands. The present study describes the baseline assessment of the public need and pre-campaign level of awareness. The target population for this study were community-dwelling people in midlife (between 40 and 75 years). The study sample was determined in two steps. First, people living in the Province of Limburg who had participated in a previous national health survey (*Gezondheidsmonitor 2016*) from the municipal health services (*GGD*) and who agreed to be contacted for future studies were invited to participate (hereafter: the ‘provincial sample’). From this total sample, a random number of 711 individuals aged 40–75 years old, stratified by region (North and South Limburg), were invited by email to participate. Because *MijnBreincoach* consists of both a mass media approach, aimed at the total Province, as well as a community-participation approach to prevention, a random sample of 629 individuals within the relevant age range from three “living labs” in the towns of Brunssum, Landgraaf and Roermond were invited to participate in a second step (hereafter: the ‘district sample’). For this, a random selection based on ZIP codes and age (40–75 years) was drawn by the *GGD* (South Limburg) or the municipality (North Limburg) from the municipal register of the three districts. The three districts were chosen to allow for variation in average neighbourhood socioeconomic status. The Ethics Review Committee Psychology and Neuroscience (ERCPN) of Maastricht University approved this study (reference number 177_07_03_2017).

### Measurements

All participants received an invitational e-mail (provincial sample) or letter (district sample) with a unique login code to complete an online informed consent form followed by the actual questionnaire using Qualtrics survey software. The socio-demographic variables gender, age, marital status and level of education were included in the questionnaire. Level of education was obtained by self-assessment of the highest finalized degree and categorized into low (primary school or low vocational education), middle (intermediate secondary education or intermediate vocational or higher secondary education) and high (higher vocational education or university). To assess general dementia literacy, we used ten translated items from the BSA survey of the UK [2]. These items concerned self-reported knowledge of dementia, personal experience with people with dementia, dementia risk awareness and knowledge of five modifiable dementia risk and protective factors (hypertension, smoking, physical activity, depression and diabetes mellitus). We included seven additional modifiable risk and protective factors (obesity, coronary heart disease, chronic kidney disease, hypocholesteraemia, mental activity, low to moderate alcohol intake and healthy diet), in order to assess all the 12 modifiable risk and protective factors included in the “LIfestyle for BRAin Health” (LIBRA) score [21, 24, 25]. In addition, four sham factors were included (use of painkillers, exposure to ambient noise, personal hygiene and having children) to check for monotone answering tendency. Additional items were developed to evaluate the needs, wishes and barriers of participants concerning brain health, such as the need for further information, preferred information source, subjective barriers to engage in a brain-healthy lifestyle, and motivation to use an internet application to increase risk factor awareness. The total questionnaire consisted of 31 items, with two additional follow-up items for participants who stated to be interested in using an e-Health platform concerning brain health. Most items were set up as statements. Participants were asked to what extent they agreed or disagreed on a 5-point Likert scale ranging from ‘strongly agree’ to ‘strongly disagree’. An English translation of the complete survey is appended in an additional file (Additional file 1).

### Statistical analysis

χ^2^ tests were used to examine whether the demographic variables age group (< 65 years and ≥ 65 years), sex, marital status and educational level were associated with level of awareness, knowledge of risk and protective factors and needs, wishes and barriers. All analyses were done in Stata 13.1 (StataCorp, College Station, TX, USA), and the level of statistical significance used was *p* < 0.05 in two-tailed tests.

## Results

### Demographics

A flowchart of the recruitment process has been added for both the provincial and the district sample as two additional files (Additional files 2 and 3). For the provincial sample, 381 (53.6%) of the 711 invited individuals participated, and 209 (33.2%) out of 629 for the district sample. In the latter, the response rate of Brunssum was lower (25.7%) than of Landgraaf (35.7%) and Roermond (37.1%). The characteristics of the provincial and district sample are presented in Table 1, and the characteristics of the three districts are summarized in Table 2. Some sample differences in demographic variables were observed. Compared to the provincial sample, participants of the three districts had a significantly lower level of education (χ^2^ (2) = 29.57, *p* = <.001). When analysing the three districts separately, it appeared that participants of the district of Brunssum were older, with a higher proportion of people aged 65 years and older (χ^2^ (2) = 18.03, *p* = <.001), and had significantly lower levels of education than participants of Roermond (χ^2^ (1) = 4.17, *p* = .041) and Landgraaf (χ^2^ (1) = 4.17 *p* = .041).

**Table 1:** Characteristics of the provincial sample and the district sample

Sample characteristics	Province of Limburg (*N* = 381)	Districts (*N* = 209)
Age, mean (SD)	61.1 (8.9)	60.1 (8.6)
Age group (year), *n* (%)		
40–50	52 (13.7%)	33 (15.9%)
51–60	115 (30.2%)	72 (34.6%)
61–70	155 (40.7%)	78 (37.5%)
71–75	59 (15.5%)	25 (12%)
Female gender, *n* (%)	164 (44%)	105 (50%)
Marital status, *n* (%)		
Married or living together	299 (79.5%)	172 (82.3%)
Not or never been married	18 (4.8%)	12 (5.7%)
Divorced	33 (8.8%)	15 (7.2%)
Widowed	26 (6.9%)	10 (4.8%)
Educational level^a^, *n* (%)		
Low	46 (12.2%)	55 (26.3%)***
Middle	134 (35.6%)	88 (42.1%)
High	196 (52.1%)	66 (31.6%)

Note: ^a^Level of education was self-reported and categorized as follows: low (primary school or low vocational education), middle (intermediate secondary education or intermediate vocational or higher secondary education) and high (higher vocational education or university). *** *p* < 0.001

**Table 2:** Characteristics of the three districts

Sample characteristics	Roermond (*N* = 78)	Landgraaf (*N* = 75)	Brunssum (*N* = 56)
Age, mean (SD)	56.9 (8.7)	60.9 (7.7)	63.3 (8.1)***
Age group (year), *n* (%)			
40–50	19 (24.4%)	8 (10.8%)	6 (10.7%)
51–60	31 (39.7%)	26 (35.1%)	15 (26.8%)
61–70	24 (30.8%)	31 (41.9%)	23 (41.1%)
71–75	4 (5.1%)	9 (12.2%)	12 (21.4%)
Female gender, *n* (%)	39 (50%)	38 (51%)	28 (50%)
Marital status, *n* (%)			
Married or living together	66 (85%)	64 (85%)	42 (75%)
Not or never been married	4 (5%)	4 (5%)	4 (7%)
Divorced	5 (6%)	5 (7%)	5 (9%)
Widowed	3 (4%)	2 (3%)	5 (9%)
Educational level^a^, *n* (%)			
Low	18 (23%)	15 (20%)	22 (39%)*
Middle	32 (41%)	33 (44%)	23 (41%)
High	28 (36%)	27 (36%)	11 (20%)

Note: ^a^Level of education was self-reported and categorized as follows: low (primary school or low vocational education), middle (intermediate secondary education or intermediate vocational or higher secondary education) and high (higher vocational education or university). *** *p* < 0.001, * *p* < 0.05

### Dementia literacy

Of the total sample, 44% of the respondents (*n* = 254) were aware of a relationship between brain health and lifestyle by stating that dementia risk reduction is possible. People with a lower level of education (χ^2^ (2) = 53.46, *p* < .001) and people aged 65 years and older (χ^2^ (1) = 9.12, *p* < .01) were less likely to agree that dementia risk reduction is possible. No differences were found between men and women (χ^2^ (1) = 0.42, *p* = .517), the provincial sample and the district sample (χ^2^ (1) = 2.78, *p* = .10), and between districts (χ^2^ (2) = 1.47, *p* = .480).

### Knowledge on dementia risk and protective factors

**Fig. 1:**
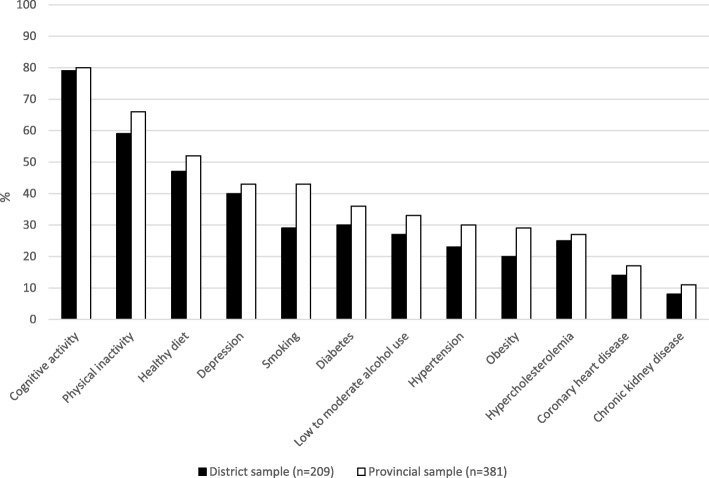
Identified risk and protective factors for the provincial sample and the district sample

**Fig. 2:**
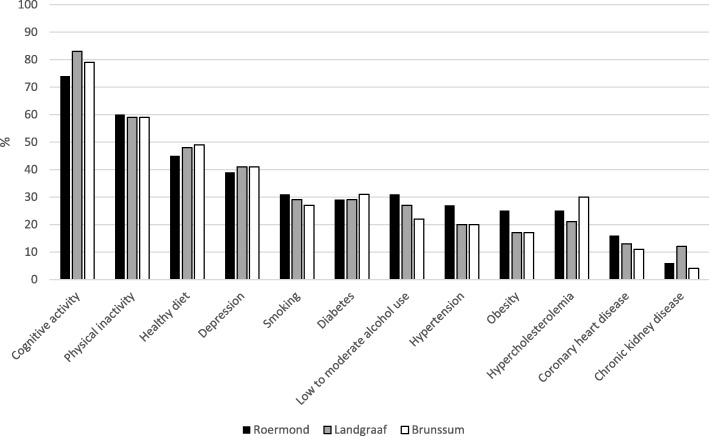
Identified risk and protective factors for the three districts

**Fig. 3:**
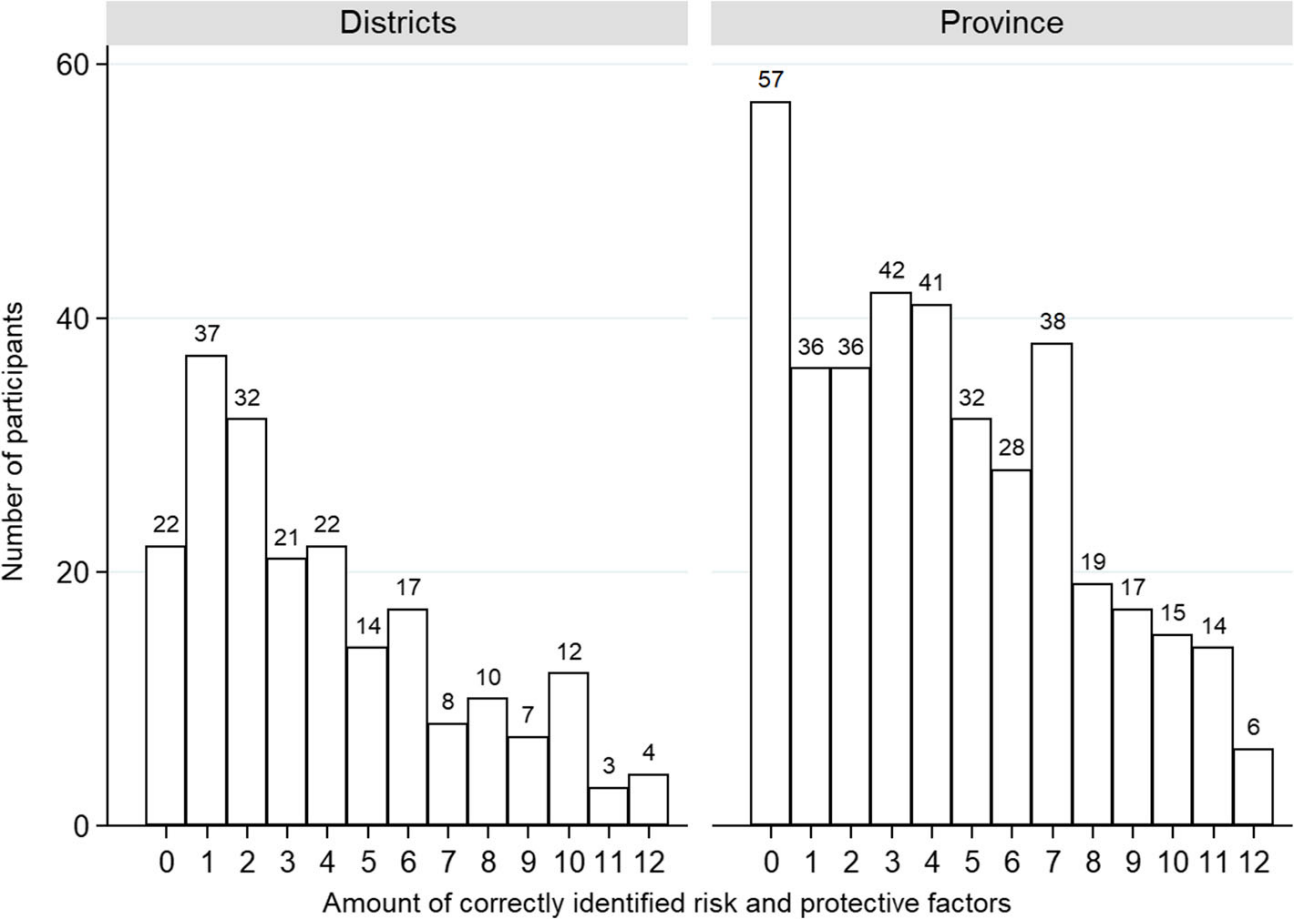
Amount of correctly identified risk and protective factors for the provincial sample and the district sample

**Fig. 4:**
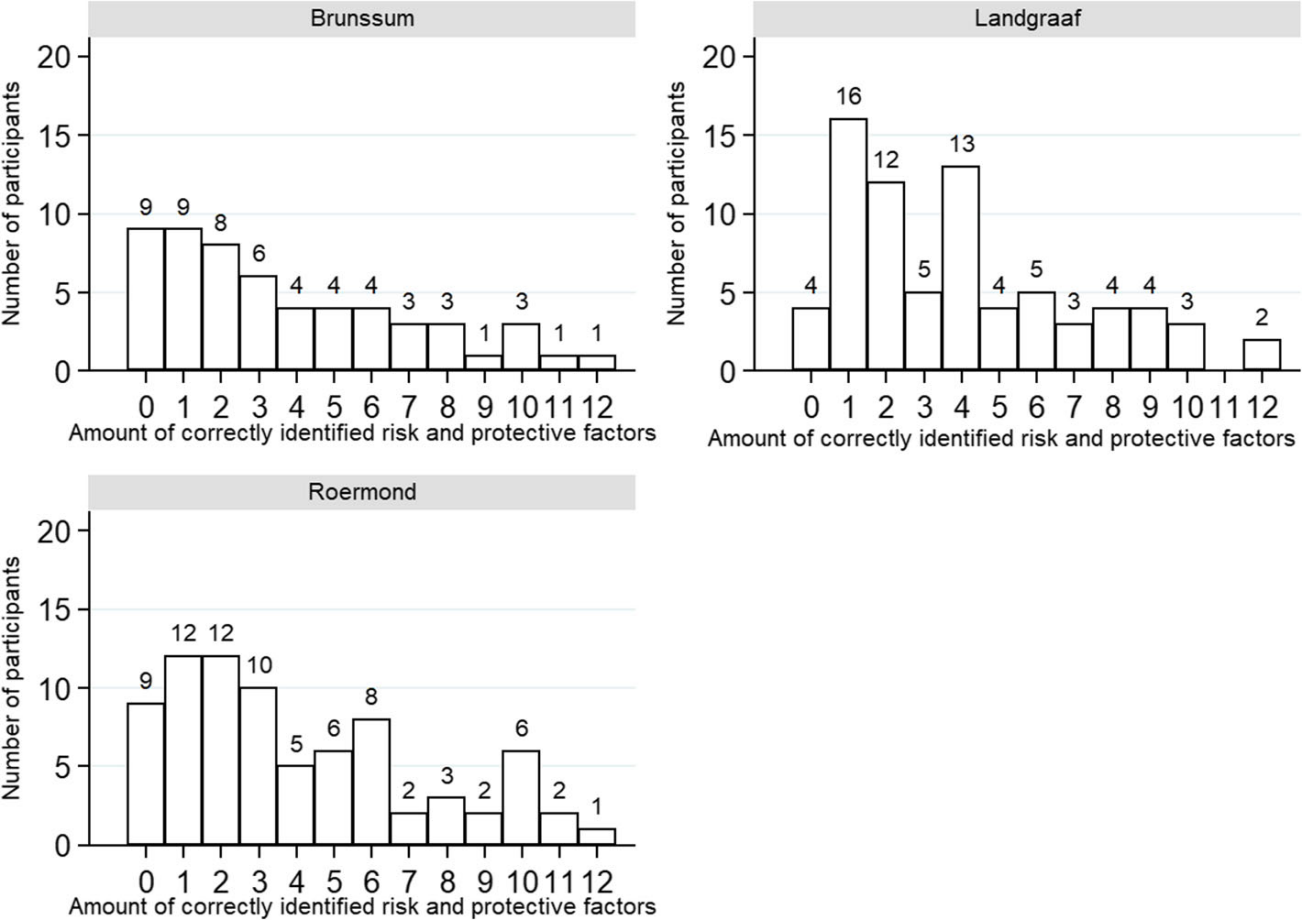
Amount of correctly identified risk and protective factors for the three districts seperately

More than half of the total sample (59%) identified zero to four of the twelve factors, with more than 10% unable to recognize any of them and only 1.7% identifying all factors correctly. Figure 1 presents the percentage of identified dementia risk and protective factors for the provincial sample and the district sample. Figure 2 displays a comparison between the three districts. Figures 3 and 4 give an overview of the amount of correctly identified risk and protective factors in both the provincial and district sample and for the three districts separately. In both the provincial and the district sample, a cognitively active lifestyle was identified most often (province 80%; districts 79%), followed by physical activity (province 66%; districts 59%) and a healthy diet (province 52%; districts 47%). Vascular factors such as hypertension (province 30%; districts 23%), hypercholesterolemia (province 27%; districts 25%), coronary heart disease (province 17%; districts 14%) and chronic kidney disease (province 11%; districts 8%) were identified least often. With regard to the sham factors (use of painkillers, exposure to ambient noise, personal hygiene and having children), more than 90% of the participants correctly rejected them as valid risk factors for dementia. Significantly fewer risk and protective factors were identified by participants with lower education (χ^2^ (12) = 35.94, *p* = <.001), except for the risk factors depression (χ^2^ (1) = 2.46, *p = .117)*, hypercholesterolemia (χ^2^ (1) = 1.79, *p = .181)* and coronary heart disease (χ^2^ (1) = 3.46, *p = .063)*. Participants who stated that their level of knowledge concerning dementia was excellent, good or considerable identified significantly more risk and protective factors than participants stating that their knowledge was poor (χ^2^ (12) = 28.50, *p* = <.01).

### Needs, wishes and barriers

The majority of participants (*n* = 382, 70%) stated they would like to receive more information about the relationship between lifestyle and brain health. Most people preferred information by searching on the web (province 61%; districts 56%), followed by visiting the website of the Dutch Alzheimer’s Association (province 36%; districts 39%), consulting their general practitioner (province 36%; districts 43%), visiting the website of the municipal health services (province 14%; districts 19%) and visiting the library (province 5%; districts 3%). People from the provincial sample (χ^2^ (2) = 17.27, *p* = <.001) and people with a higher level of education (χ^2^ (4) = 11.96, *p* < .05) were more likely to request further information. Fifty-four percent (*n* = 291) of the participants stated they would like to use an Internet application in order to learn more about improving their brain health, with another 27% (*n* = 147) willing to consider it. No statistical differences with regard to the use of an Internet application were observed between the provincial and district sample (χ^2^ (2) = 0.06, *p = 0.971*) nor for level of education (χ^2^ (4) = 4.93, *p = 0.294*) and sex (χ^2^ (2) = 5.65, *p* = 0.06). The majority of participants aged 65 years old or above would use, or consider using, an Internet application (*n* = 176, 81%), which was not significantly different (χ^2^ (2) = 2.92, *p* = .232) from younger ages (*n* = 266, 81%). The largest barrier for adopting a brain-healthy lifestyle was stated to be lack of knowledge (province 42%; districts 38%), followed by lack of motivation (province 17%; districts 13%) and lack of time (province 14%; districts 11%). Other barriers were difficulty organizing (province 8%, districts 5%), financial reasons (province 4%; districts 6%), health problems (province 4%; districts 2%) and ‘other reasons’ (province 4%, districts 3%).

## Discussion

This study assessed dementia literacy and knowledge concerning dementia risk and protective factors in middle-aged and older individuals living in the community. Results clearly showed that the majority of individuals were unaware of the relationship between lifestyle-related risk and protective factors and brain health. Considerable gaps in knowledge exist regarding common dementia risk factors such as hypertension, hypercholesterolemia and coronary heart disease. Most people were eager to receive more information on the topic of brain health and most participants were also positive about using eHealth tools to improve brain health. Variation in level of awareness by level of education and age was observed.

The results of this study are in line with previous studies reporting on the level of awareness of the relationship between lifestyle and brain health and risk and protective factors for dementia [2, 22, 23]. The methodology of this study is comparable to the BSA study in the UK, and therefore it is noteworthy that the UK and Dutch population show very similar levels of awareness, with less than half of the participants (47 and 44%, respectively) reporting that the risk for dementia can be influenced [2]. As for the identification of lifestyle factors, our study is also congruent with the findings of a recent systematic review examining population surveys concerning the public’s knowledge and understanding of dementia [22]. Cognitive activity was identified most often as a protective factor [22]. Despite the good evidence for cardiovascular risk factors such as hypertension, hypercholesterolemia and diabetes [12, 15, 26, 27], only few people seem aware that “what is good for your heart is good for your brain”.

Together, these studies make a strong case for informing the public more effectively about modifiable dementia risk and protective factors. The report of the National Academies of Sciences, Engineering and Medicine (NASEM) from 2017 states that evidence for risk and protective factors of brain health is still inconclusive, yet compelling. The report stresses the importance of providing the public with easily accessible information about the effect of targeting those risk factors that are supported by promising research, i.e. cognitive activity, physical activity and adequate blood pressure control [12]. The Lancet Commission on Dementia Prevention, Intervention and Care underlines this by recommending ‘to be ambitious about dementia prevention’. The Commission underscores that society as a whole has a responsibility to not only provide information about dementia prevention, but also implement low-level interventions [4].

It seems that people with a lower socioeconomic status and older people are more difficult to reach and motivate in eHealth and preventative campaigns [28–32]. This was also seen in our study, in which the subgroups with significantly lower dementia literacy (i.e. people with lower levels of education) were less inclined to receive further information. A one-size-fits-all approach might therefore not be efficient with regard to prevention strategies. Indeed, recent literature showed that targeting specific subgroups and engagement of local partners is crucial for the reach and effect of a prevention campaign [32].

The current findings serve as a baseline for a dementia awareness campaign that has been launched recently in the Province of Limburg, The Netherlands, called *MijnBreincoach* (“MyBraincoach”) [33]. As part of this campaign, an eHealth platform has been developed aimed at giving people insight into their own lifestyle profile and pointing them towards individual ‘room for improvement’. To investigate differences in preventive strategies, the approach is two folded. On the one hand, it targets the whole Province of Limburg through mass media (e.g. interviews in newspapers and TV), posters and flyers in public spaces and involvement of cultural and health-related authorities. On the other hand, a district-oriented campaign implements measures tailored towards the needs and wishes of the three districts that were part of our survey. The latter approach was developed together with the local community, local professionals and health care providers, offering a variety of activities (e.g. lectures in community centres, training to local health care providers, involvement of small and medium-sized enterprises).

Strengths of our study include the use of a comparable methodology as previous dementia literacy studies [2] in order to add up to the existing evidence. Our study has, however, some limitations. First, selection bias may have occurred in general since non-Dutch speakers were excluded, which likely excludes minority groups who are less educated and health literate. Second, the potential for the use of Internet applications found in our study could be an overestimation since our study design consisted mainly of online questionnaires. Third, it might be that the question whether people can do something to reduce their dementia risk was misunderstood by some participants. For instance, older individuals (65–75 years) might be more likely to relate this question to their own resources and possibilities in comparison with peers, rather than speculating on the modifiability of dementia risk through lifestyle changes in general. Unfortunately, we do not have any data on why people answered in a certain fashion. However, these people, irrespective of the reason for their answer, belong to the same target group in whom awareness can be increased as there is still room for improvement in this age group in terms of cognitive health as shown by previous studies using the LIBRA index [18, 20, 21]. Furthermore, the provincial sample was composed of participants from the *Gezondheidsmonitor 2016* who agreed to participate in further research. This pre-selection of people willing to participate in scientific research might not be an exact reflection of the general population, due to factors such as level of education and general health knowledge, which might lead to an overestimation of awareness levels. In addition, we could not compare the demographics of those who did and did not take part in this study because linkage of the two surveys (datasets) was only possible in those that did consent to participate in the present study. Still, our findings are comparable to the results of the BSA study in the UK. Also, differences between the provincial and district samples were minimal. Regarding the latter, the Brunssum district showed the lowest level of awareness and the lowest level of education. It should be noted that this district was selected a priori because of its known, relatively low socioeconomic status.

## Conclusions

Our study corroborates the evidence that the majority of people in the general population are unaware of a relationship between lifestyle-related factors and brain health, and most people in this study expressed a need for brain-health education. Major gaps in knowledge exist in particular amongst the cardiovascular risk factors for dementia. These findings stress the importance of informing the public about lifestyle related risk and protective factors of brain health and dementia via health promotion campaigns.

## Additional files

Additional file 1: English language translation of the survey. (DOCX 17 kb)

Additional file 2: Flowchart of the recruitment process of the provincial sample. (JPG 52 kb)

Additional file 3: Flowchart of the recruitment process of the district sample. (JPG 125 kb)

## Data Availability

The datasets used and/or analysed during the current study are available from the corresponding author on reasonable request.

## Abbreviations

BSA: British Social Attitudes
ERCPN: Ethics Review Committee Psychology and Neuroscience
*GGD*: Municipal Health Services
LIBRA: LIfestyle for BRAin Health
NASEM: National Academies of Sciences, Engineering and Medicine
UK: United Kingdom
