# Supplementary material for: Dementia awareness and risk perception in middle-aged and older individuals: baseline results of the MijnBreincoach survey on the association between lifestyle and brain health
Source: BMC Public Health. 2019 Jun 3;19:678. doi: 10.1186/s12889-019-7010-z (PMC6545627; doi:10.1186/s12889-019-7010-z)
Supplement: Supplementary file 1 — English language translation of the survey. (DOCX 17 kb) [file 12889_2019_7010_MOESM1_ESM.docx]

**Dementia awareness questionnaire**

-------------------------------------------------------------------------------------------------------------------------------

Demographics

1. How old are you?
   *[textfield for number between 40 and 75]*
2. What is your gender?
   - Male
   - Female
3. What is your zip code?
   *[textfield of four numbers]*
4. What is your marital status?
   - Married/registered partnership
   - Living together
   - Unmarried, never been married
   - Divorced
   - Widowed
5. What is your highest finalized degree of education?
   *[Six categories according to the Dutch education system, categorized into low, medium and high]*

------------------------------------------------------------------------------------------------------------------------------------------

Dementia knowledge

1. Dementia describes a set of symptoms including loss of memory, sudden mood swings, not remembering who people are, and having trouble finding your words. Alzheimer's disease is one form of dementia. How much would you say you know about dementia?
   - A great deal
   - Quite a lot
   - Some
   - Not very much
   - Nothing at all
   - I don't know
   - I prefer not to answer this question
2. Have you ever personally known anyone with dementia or have it yourself?
   [*Please choose all that apply*]
   - No, I don't know anyone who has, or had, dementia
   - Yes, my job involves / involved working with people who have dementia
   - Yes, I have dementia myself
   - Yes, my partner, parents (in-law), of children
   - Yes, my grandparents of brother/sister
   - Yes, a friend(s) I know fairly well
   - Yes, a friend(s) or acquaintance(s) I know less well
   - Yes, a colleague / someone at my work
   - Yes, someone else
   - I am not sure
   - I prefer not to answer this question
3. Leaving aside anything you have done as part of your job, please say which, if any, of the following you have done for someone with dementia, either now or in the past?

[*Please choose all that apply*]

- Cared for someone (other than myself) who lives / lived with me
- Cared on a regular basis for someone who does not / did not live with me
- Been responsible for making arrangements for someone to be looked after (e.g. arranged for a carer to visit someone at home, or arranged for someone to go into a residential home)
- Helped someone from time-to-time
- Encouraged someone showing symptoms of dementia to seek professional help
- Visited someone every now and then
- Other [*textfield*]
- None of these
- I prefer not to answer this question

------------------------------------------------------------------------------------------------------------------------------------------

Dementia risk awareness

Ple*ase state how much you agree or disagree with the following statements.*

1. ‘There is nothing anyone can do to reduce their risks of getting dementia’
   - Agree strongly
   - Agree
   - Neither agree nor disagree
   - Disagree
   - Disagree strongly
2. 'High blood pressure increases your chances of getting dementia'
   - Agree strongly
   - Agree
   - Neither agree nor disagree
   - Disagree
   - Disagree strongly
3. 'Having a parent with dementia increases the chances of getting dementia'
   - Agree strongly
   - Agree
   - Neither agree nor disagree
   - Disagree
   - Disagree strongly
4. ‘The use of painkillers increases your chances of getting dementia’
   - Agree strongly
   - Agree
   - Neither agree nor disagree
   - Disagree
   - Disagree strongly
5. ‘Smoking increases your chances of getting dementia’
   - Agree strongly
   - Agree
   - Neither agree nor disagree
   - Disagree
   - Disagree strongly
6. ‘No or moderate alcohol use lowers your chances of getting dementia’
   - Agree strongly
   - Agree
   - Neither agree nor disagree
   - Disagree
   - Disagree strongly
7. Regular physical activity lowers your chances of getting dementia’
   - Agree strongly
   - Agree
   - Neither agree nor disagree
   - Disagree
   - Disagree strongly
8. ‘Working in a noisy environment increases your chances of getting dementia’
   - Agree strongly
   - Agree
   - Neither agree nor disagree
   - Disagree
   - Disagree strongly
9. ‘Depression increases the chances of getting dementia’
   - Agree strongly
   - Agree
   - Neither agree nor disagree
   - Disagree
   - Disagree strongly
10. ‘Diabetes increases the chances of getting dementia’
    - Agree strongly
    - Agree
    - Neither agree nor disagree
    - Disagree
    - Disagree strongly
11. ‘Being overweight increases the chances of getting dementia’
    - Agree strongly
    - Agree
    - Neither agree nor disagree
    - Disagree
    - Disagree strongly
12. ‘Poor personal hygiene increases the chances of getting dementia’
    - Agree strongly
    - Agree
    - Neither agree nor disagree
    - Disagree
    - Disagree strongly
13. ‘A mentally active lifestyle lowers the chances of getting dementia’
    - Agree strongly
    - Agree
    - Neither agree nor disagree
    - Disagree
    - Disagree strongly
14. ‘Heart disease increases the chances of getting dementia’
    - Agree strongly
    - Agree
    - Neither agree nor disagree
    - Disagree
    - Disagree strongly
15. ‘Kidney disease increases the chances of getting dementia’
    - Agree strongly
    - Agree
    - Neither agree nor disagree
    - Disagree
    - Disagree strongly
16. ‘Having children increases the chances of getting dementia’
    - Agree strongly
    - Agree
    - Neither agree nor disagree
    - Disagree
    - Disagree strongly
17. ‘High cholesterol increases the chances of getting dementia’
    - Agree strongly
    - Agree
    - Neither agree nor disagree
    - Disagree
    - Disagree strongly
18. ‘Healthy diet lowers the chances of getting dementia’
    - Agree strongly
    - Agree
    - Neither agree nor disagree
    - Disagree
    - Disagree strongly
19. Here is a list of factors that influence the risk of getting dementia, together with factors that have no influence on the risk of getting dementia. Which of the factors described below are the most important factors for the development of dementia according to you?
    [*Three options possible*]
    - High blood pressure
    - Having parents with dementia
    - Use of painkillers
    - Smoking
    - No or moderate use of alcohol
    - Regular physical activity
    - Working in a noisy environment
    - Depression
    - Diabetes
    - Obesity (being overweight)
    - Poor personal hygiene
    - A mentally active lifestyle
    - Heart disease
    - Kidney disease
    - Having children
    - High cholesterol
    - Healthy diet
20. Would you be interested in receiving information on how to improve your brain health?
    - Yes
    - No
    - Maybe
21. In the case that you would like to know more about your own brain health, what information channels would you use to find this information?
    [*Please choose all that apply*]
    - Search on the web
    - GP office
    - Webpage municipal health services
    - Website Dutch Alzheimer’s Association
    - Library
    - Other [*textfield*]
    - None of these
    - I do not know
    - I prefer not to answer this question
22. In the case that you would decide to work on improving your own brain health, what could be an obstructing factor for you?
    [*Please choose all that apply*]
    - Lack of knowledge
    - Lack of time
    - Financial reasons
    - Lack of motivation
    - Difficult to organize
    - Health problems
    - Other [*textfield*]
    - None of these
    - I do not know
    - I prefer not to answer this question
23. In the case that there was a mobile application, providing you without charge with information about your brain health en giving advice on how to improve your brain health, would you use this app?
    - Yes
    - No
    - Maybe

[*If answer is No, go to End*]

[*If answer is Yes or Maybe, go the question 27*]

1. Maastricht University has started a research line focused on prevention of dementia, in collaboration with the municipal health services. This research includes the development of an app (mobile application) that could give you more insight into your own brain health and how to improve your brain health. Would you be interested and do you give consent to be contacted for this research?
   - Yes, I give consent
   - No, I give no consent

[*If answer is No, go to End*]
[*If answer is Yes, go to question 28*]

1. Please leave the phone number and/or e-mail-address that we can use to contact you for this research.
   [*Text field*]

End

Thank you very much for filling in this questionnaire. If you have any remarks or questions regarding this study, please contact us via [*email address*].
