# Supplementary figures and images for: Dementia awareness and risk perception in middle-aged and older individuals: baseline results of the MijnBreincoach survey on the association between lifestyle and brain health
Source: BMC Public Health. 2019 Jun 3;19:678. doi: 10.1186/s12889-019-7010-z (PMC6545627; doi:10.1186/s12889-019-7010-z)

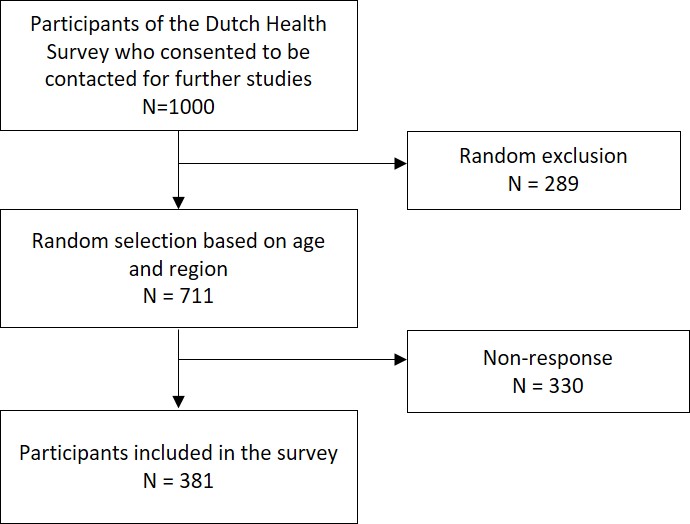

Supplement: Supplementary file 2 — Flowchart of the recruitment process of the provincial sample. (JPG 52 kb) [file 12889_2019_7010_MOESM2_ESM.jpg]

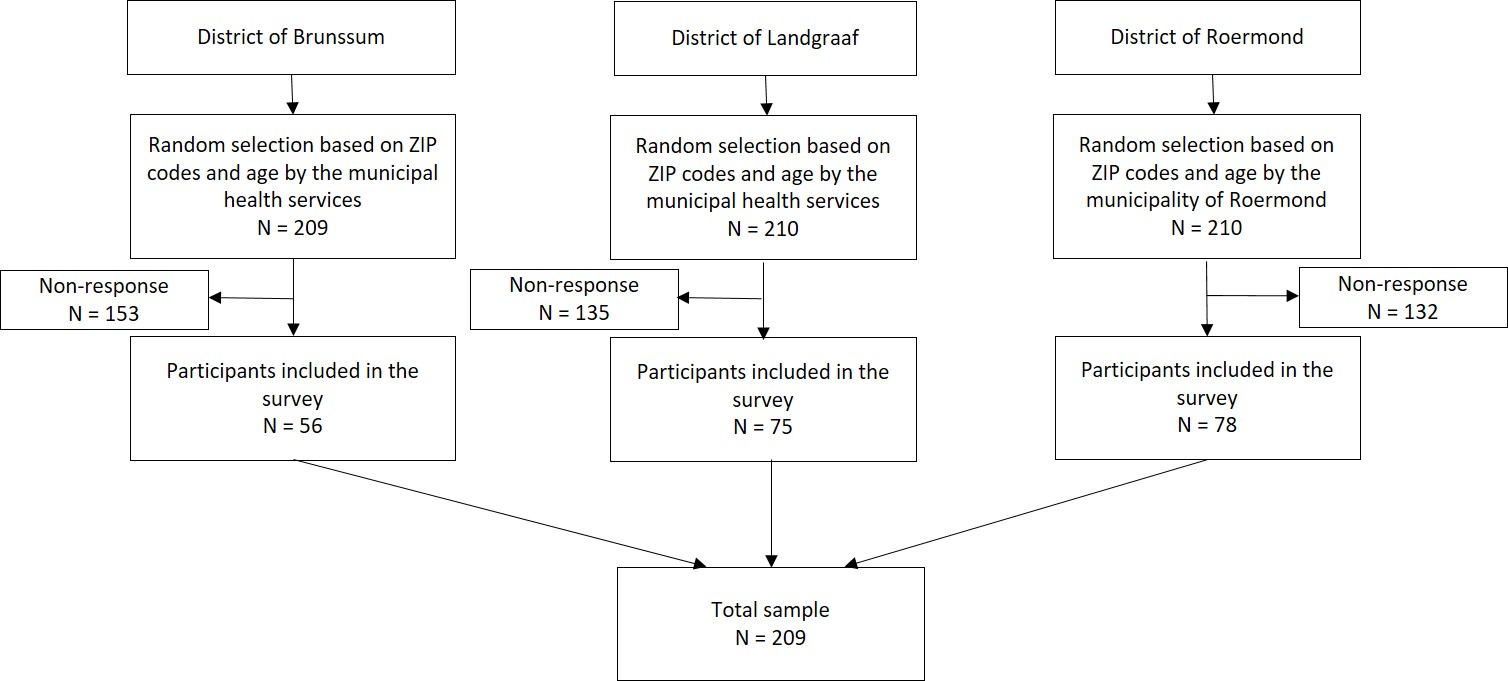

Supplement: Supplementary file 3 — Flowchart of the recruitment process of the district sample. (JPG 125 kb) [file 12889_2019_7010_MOESM3_ESM.jpg]
